# A product of its environment: the epaulette shark (*Hemiscyllium ocellatum*) exhibits physiological tolerance to elevated environmental CO_2_

**DOI:** 10.1093/conphys/cou047

**Published:** 2014-10-15

**Authors:** Dennis D. U. Heinrich, Jodie L. Rummer, Andrea J. Morash, Sue-Ann Watson, Colin A. Simpfendorfer, Michelle R. Heupel, Philip L. Munday

**Affiliations:** 1 School of Marine and Tropical Biology, James Cook University, Townsville, Queensland 4811, Australia; 2 ARC Centre of Excellence for Coral Reef Studies, James Cook University, Townsville, Queensland 4811, Australia; 3 University of Tasmania, Institute for Marine and Antarctic Studies (IMAS), Sandy Bay, Tasmania 7001, Australia; 4 Centre for Sustainable Tropical Fisheries and Aquaculture, School of Earth and Environmental Science, James Cook University, Townsville, Queensland 4811, Australia; 5 Australian Institute of Marine Science, Townsville, Queensland 4810, Australia

**Keywords:** Climate change, ecophysiology, elasmobranch, hypoxia tolerance, ocean acidification

## Abstract

Ocean acidification, resulting from increasing anthropogenic CO_2_ emissions, is predicted to affect the physiological performance of many marine species. Recent studies have shown substantial reductions in aerobic performance in some teleost fish species, but no change or even enhanced performance in others. Notably lacking, however, are studies on the effects of near-future CO_2_ conditions on larger meso and apex predators, such as elasmobranchs. The epaulette shark (*Hemiscyllium ocellatum*) lives on shallow coral reef flats and in lagoons, where it may frequently encounter short-term periods of environmental hypoxia and elevated CO_2_, especially during nocturnal low tides. Indeed, *H. ocellatum* is remarkably tolerant to short periods (hours) of hypoxia, and possibly hypercapnia, but nothing is known about its response to prolonged exposure. We exposed *H. ocellatum* individuals to control (390 µatm) or one of two near-future CO_2_ treatments (600 or 880 µatm) for a minimum of 60 days and then measured key aspects of their respiratory physiology, namely the resting oxygen consumption rate, which is used to estimate resting metabolic rate, and critical oxygen tension, a proxy for hypoxia sensitivity. Neither of these respiratory attributes was affected by the long-term exposure to elevated CO_2_. Furthermore, there was no change in citrate synthase activity, a cellular indicator of aerobic energy production. Plasma bicarbonate concentrations were significantly elevated in sharks exposed to 600 and 880 µatm CO_2_ treatments, indicating that acidosis was probably prevented by regulatory changes in acid–base relevant ions. Epaulette sharks may therefore possess adaptations that confer tolerance to CO_2_ levels projected to occur in the ocean by the end of this century. It remains uncertain whether other elasmobranchs, especially pelagic species that do not experience such diurnal fluctuations in their environment, will be equally tolerant.

## Abstract (precis)

Ocean acidification is predicted to affect the performance of marine species, but little is known about the effects on sharks. We found that long-term exposure to elevated CO_2_ did not affect the epaulette shark, possibly because it experiences fluctuating environmental conditions in its shallow coral reef habitat.

## Introduction

Anthropogenic CO_2_ emissions have caused an increase in atmospheric CO_2_ by almost 40% over the past 250 years (IPCC, 2013). The resulting rise from pre-industrialization levels (∼280 ppm) to 400 ppm in 2014 has occurred at a rate unprecedented for the past 800 000–1 000 000 years (Raven *et al.*, 2005; Doney and Schimel, 2007; Lüthi *et al.*, 2008). The oceans have absorbed more than 30% of the additional CO_2_ released by human activities, thus tempering the atmospheric rise in CO_2_ (Sabine *et al.*, 2004; Sabine and Feely, 2007). However, the resulting rise in seawater CO_2_ partial pressure (PCO_2_) and the associated reduction in pH, called ocean acidification, is a significant threat to marine organisms and ecosystems (Hoegh-Guldberg *et al.*, 2007; Fabry *et al.*, 2008).

The reduced carbonate saturation state that accompanies lower seawater pH affects the ability of calcifying marine organisms to form carbonate shells and skeletons (Orr *et al.*, 2005; Doney *et al.*, 2009), but rising oceanic CO_2_ may also impact the respiratory physiology of many water-breathing organisms. Acid–base disturbances related to elevated environmental CO_2_ can reduce oxygen uptake and delivery, which could directly impact metabolic performance. Reductions in an organism's scope for aerobic metabolic performance can result in less energy being available for crucial life-history processes, such as growth and reproduction (Pörtner, 2008; Pörtner and Farrell, 2008). For instance, Humboldt squid (*Dosidicus gigas*) exhibit a 30% reduction in resting metabolic rate and a 45% decrease in activity upon exposure to projected near-future CO_2_ levels, owing to an impaired oxygen transport system, which would be predicted to reduce overall performance and compress their habitable depth range (Rosa and Seibel, 2008). In contrast, teleost fishes are expected to be physiologically well equipped to compensate pH and ion disturbances caused by high CO_2_ (Ishimatsu *et al.*, 2008; Brauner and Baker, 2009). Nevertheless, interspecific variation is evident in the physiological responses of teleost fish to elevated CO_2_; for example, some fishes exhibit no change in aerobic scope in high-CO_2_ environments (Ishimatsu *et al.*, 2008; Melzner *et al.*, 2009a; Couturier *et al.*, 2013), whereas others reduce aerobic scope (Munday *et al.*, 2009) and some even increase aerobic scope (Couturier *et al.*, 2013; Rummer *et al.*, 2013a) when exposed to near-future CO_2_ levels. Consequently, the effects of ocean acidification on a broad range of species, including vulnerable and tolerant species, should be investigated in order to identify traits that will be important for individual performance and success in near-future oceans and predict changes in community structure (Melzner *et al.*, 2009b).

In contrast to the growing body of knowledge about the effects of ocean acidification on teleost fishes, little is known about the impacts of rising levels of oceanic CO_2_ on elasmobranchs. Elasmobranchs buffer a pH disturbance, such as that associated with exposure to high CO_2_, in a manner similar to teleosts. Bicarbonate is accumulated in the blood, but in addition, elasmobranchs may also increase branchial ammonia excretion rates to ameliorate the acidosis further (Evans, 1982; King and Goldstein, 1983; Claiborne and Evans, 1992; Brauner and Baker, 2009; Tresguerres *et al.*, 2010). The haemoglobin of elasmobranchs also has a much higher buffering capacity compared with that of most teleosts, and thus, O_2_ transport and aerobic performance may be less sensitive to pH disturbances (Berenbrink *et al.*, 2005). Yet, it is thought that the resilience of elasmobranchs to acid–base disturbances is related largely to their sophisticated acid excretion processes at the gill (Wood *et al.*, 1995). If elasmobranchs are notably tolerant to near-future CO_2_ conditions, this could potentially increase predation pressure and alter species compositions of marine environments.

The epaulette shark (*Hemiscyllium ocellatum*) exhibits exceptionally high tolerance to the severe hypoxia (low oxygen) that it routinely experiences while inhabiting shallow coral reef flats (Routley *et al.*, 2002; Nilsson and Renshaw, 2004), and thus, it may not be surprising if this species is also tolerant to near-future CO_2_. However, acute responses may differ dramatically from the responses to long-term exposure; studies on *H. ocellatum* in response to anoxia or hypoxia have been following only minutes to hours of exposure (Wise *et al.*, 1998; Renshaw *et al.*, 2002; Routley *et al.*, 2002; Chapman and Renshaw, 2009; Dowd *et al.*, 2010; Speers-Roesch *et al.*, 2012a). No study, to date, has examined how *H. ocellatum* responds to prolonged exposure to elevated CO_2_. Given that increased uptake of CO_2_ by the ocean will affect both the average CO_2_ levels and the magnitude of extreme CO_2_ levels (Shaw *et al.*, 2013), it is important to consider longer-term responses to elevated CO_2_ beyond those that would be experienced on a diurnal basis (e.g. hours; Ohde and van Woesik, 1999; Compagno, 2002; Last and Stevens, 2009; Shaw *et al.*, 2013). Thus, both the physiological sensitivity of the organism and the variations it may already be experiencing in its habitat are important when considering which species will exhibit positive or negative responses to rising ocean CO_2_ levels. However, it is also important to consider the relationship between environmental cues and other traits, such as behaviour—which is especially relevant to species like *H. ocellatum*—when considering the importance of phenotypic plasticity, because this could influence selection over the longer term (Marais and Chown, 2008).

We exposed *H. ocellatum* to near-future CO_2_ conditions for a minimum of 60 days and measured resting oxygen consumption rates and critical oxygen tensions as proxies for resting metabolic rate and sensitivity to hypoxia, respectively. In addition to whole-organism responses, we also measured or calculated haematological and tissue parameters, including plasma ionic (HCO_3_^−^, Cl^−^, Na^+^ and K^+^) and urea concentrations, haemoglobin (Hb), mean cell haemoglobin concentration (MCHC), haematocrit (Hct), spleen–somatic index (SSI) and citrate synthase activity in heart, brain and red muscle. The aim was to provide insight into the physiological parameters that may underpin changes in metabolic performance and sensitivity to hypoxia in this species. We hypothesized that *H. ocellatum* can physiologically tolerate elevated CO_2_ because it routinely experiences daily reductions in environmental O_2_ (Routley *et al.*, 2002; Nilsson and Renshaw, 2004) and probably elevations in CO_2_. However, if CO_2_ tolerance is related to the diurnal patterns this species already experiences in their natural habitat, prolonged exposure (60 days) to elevated CO_2_ may negatively affect metabolic rate and hypoxia tolerance.

## Materials and methods

### Experimental animals

Epaulette sharks (*Hemiscyllium ocelatum*) were collected from the northern Great Barrier Reef by Northern Barrier and Cairns Marine (Cairns, Queensland, Australia) and transported to James Cook University (JCU). Five to six individuals were placed in each of six 700 l tanks in a recirculating seawater system. Individuals were measured [standard length, 33.38 ± 7.29 cm (mean ± SD)] to ensure an equal distribution of sizes among tanks. Unique fin clips along the margins of pectoral, pelvic and dorsal fins were used for individual identification. Shelter was provided in the form of PVC pipe sections placed within each tank. Food was provided once every 24 h and consisted of 4% of shark biomass per tank in raw prawn meat. There was no indication that any individuals or treatment groups were eating less than this amount throughout the duration of the study. Sharks were acclimated to laboratory conditions for at least 4 weeks prior to commencing CO_2_ treatments.

### Experimental CO_2_ conditions

The experimental system comprised three 8000 l recirculating seawater systems, each set to simulate one of the following three CO_2_ treatments: control (∼390 µatm); medium (∼600 µatm); and high (∼880 µatm). Carbon dioxide levels were achieved and maintained by CO_2_ infusion of seawater in 3000 l sumps attached to each recirculating seawater system. The pH_NBS_ (National Bureau of Standards scale) levels were set to match target CO_2_ concentrations and maintained using a CO_2_-infusing system (Aqua Medic GmbH, Bissendorf, Germany). If the pH rose above the set point, an electronic solenoid initiated the system to deliver a steady stream of CO_2_ into a diffuser within the corresponding sump. Carbon dioxide-equilibrated seawater from each system was delivered to two replicate 700 l tanks (∼25 l min^−1^) per treatment. Each tank contained five or six sharks, as described above. This central approach of pH manipulation allowed for stability in seawater pH and PCO_2_ within the holding tanks. Tanks were covered with transparent plastic sheeting to reduce CO_2_ loss to the atmosphere.

The pH_NBS_ was measured daily (Hach, model #HQ40d) in each tank to ensure that it remained within ±0.05 of desired levels. Temperatures were also measured daily and maintained at 28.5°C by automated heater/chillers attached to each seawater system. Salinity and alkalinity were measured on a weekly basis. Total alkalinity (TA) was estimated using Gran titrations and certified reference materials (Dr A. G. Dickson, Scripps Institution of Oceanography). Average seawater PCO_2_ was calculated using these parameters in CO2SYS (Pierrot *et al.*, 2006) and using constants from Dickson and Millero (1987) (Table 1).

**Table 1:** Mean values for PCO_2_, pH, total alkalinity, salinity and temperature over the course of the CO_2_ exposure period

Treatment	Tank number	PCO_2_ (μatm)	pH	Total alkalinity (μmol kg^−1^)	Salinity (ppt)	Temperature (°C)
Control	1	397 ± 6.5	8.16 ± 0.006	2145 ± 4.7	35.6 ± 0.07	28.6 ± 0.05
Control	2	384 ± 6.8	8.18 ± 0.006	2145 ± 4.7	35.6 ± 0.07	28.4 ± 0.04
Medium	1	614 ± 16.6	8.00 ± 0.009	2095 ± 5.1	35.9 ± 0.07	28.7 ± 0.05
Medium	2	608 ± 16.5	8.00 ± 0.009	2095 ± 5.1	35.9 ± 0.07	28.6 ± 0.05
High	1	876 ± 14.6	7.86 ± 0.006	2079 ± 5.3	36.0 ± 0.03	28.7 ± 0.03
High	2	861 ± 14.4	7.87 ± 0.006	2079 ± 5.3	36.0 ± 0.03	28.7 ± 0.04

Total alkalinity was measured weekly for each treatment condition, and temperature was measured daily for each tank within each treatment. Means were calculated for each treatment over the entire experimental period and are given for each holding tank, ±SEM. Abbreviation: PCO_2_, seawater carbon dioxide partial pressure.

Sharks were introduced to the CO_2_ treatments following 30 days acclimation to laboratory holding conditions and were then maintained in their respective CO_2_ treatment conditions for a minimum of 60 days prior to physiological experimentation.

### Experimental protocol

#### Resting oxygen consumption rates

Resting O_2_ consumption rates (*Ṁ*O_2Rest_) were determined for sharks following 60–68 days of exposure to control (*n* = 10), medium (*n* = 12) and high (*n* = 11) CO_2_ conditions and a 48 h fasting period using an intermittent-flow respirometry system with purpose-built respirometry chambers. Animals were transferred individually into the cylindrical 11 or 15 l respirometry chambers (depending on animal body size) submerged in a temperature-controlled aquarium (28.5°C) within each animal's respective experimental CO_2_ conditions and habituated to the chamber for 12 h before oxygen consumption measurements commenced. Submersible pumps were fitted to each chamber to supply a continuous water flow (1300 l h^−1^; WEIPRO WH-2000; Yongcheng Aquarium Co., Ltd, Guangdong, China) from the surrounding water bath through the chambers. During respirometry trials, a digital relay timer (MFRT-1 Multi Function Recycling Timer; Xiamen SUPERPRO Technology Co., Ltd, Xiamen, Fujian, China) was used to stop water flow for 15 or 20 min and then resume flushing for 15 min over a total period of 12 h. The intervals of interrupted water flow were short enough to ensure that oxygen within the chambers did not fall below 80% saturation at any time, while flush periods were long enough to eliminate accumulation of metabolic CO_2_ and allow water oxygen levels to return to 100% saturation (Steffensen *et al.*, 1984; Steffensen, 1989). A second pump (1300 l h^−1^; WEIPRO WH-2000) was connected to each respirometry chamber to recirculate water continuously within the chamber, thus ensuring complete mixing and homogeneous water *P*O_2_ (*P*_W_O_2_). Contactless spots (2 mm) with oxygen-sensitive REDFLASH dye were adhered to the inside of glass tubes connected to the recirculating pumps on each respirometer. These spots were then linked to a Firesting Optical Oxygen Meter (Pyro Science e. K., Aachen, Germany) via 5 m fibre-optic cables to record continuously (0.5 Hz) the temperature-compensated O_2_ concentration (in milligrams per litre) of the water within each chamber over the 12 h period of time. The 0 and 100% oxygen levels of the Firesting oxygen meter were calibrated using 0 and 100% air-saturated seawater. At the end of each trial, the wet mass was taken for each shark [232.47 ± 117.98 g (mean ± SD)] prior to release back to experimental holding conditions.

#### Critical oxygen tension

Upon completion of *Ṁ*O_2Rest_ measurements, sharks were permitted to recover in their respective CO_2_ treatment conditions for ∼3 weeks. Then, the same respirometers used to determine *Ṁ*O_2Rest_ were used to determine the critical oxygen tension (*P*_crit_) for the same sharks exposed to control (*n* = 9), medium (*n* = 12) and high CO_2_ (*n* = 10)*.* By this point, sharks would have been exposed to their respective experimental conditions for 85–92 days. Prior to measurements, sharks were fasted for 48 h before being introduced to the cylindrical 11–15 l respirometry chambers. Then, the *Ṁ*O_2_ of each animal was monitored for a minimum of 4 h using an intermittent flush cycle (15 min flush–15 min closed) so that stable *Ṁ*O_2Rest_ was achieved prior to commencing the hypoxia experiment. The respirometers were then sealed by turning off flush pumps and closing previously installed ball-valves downstream of the flush pumps. Oxygen levels in the chamber were monitored continuously (0.5 Hz) and allowed to decrease to at least 0.8 mg l^−1^ to ensure that the critical oxygen tension for each individual was recorded (based on estimates from Routley *et al.*, 2002). The changes in water pH and PCO_2_ that occur when using closed respirometry for a short period of time have been shown previously to have no effect on *P*_crit_ in fish (Henriksson *et al.*, 2008). After this oxygen concentration was achieved, the aforementioned flush cycle was reinstated such that O_2_ levels within each respirometer could quickly return to normoxic conditions (100% air-saturated seawater).

#### Haematological and tissue analyses

Following *P*_crit_ measurements, animals were returned to their treatment tanks to recover for ∼1 week. After this time, blood was sampled from sharks exposed to control (*n* = 8), medium (*n* = 10) and high (*n* = 8) CO_2_ conditions by inserting a 23 gauge needle posterior to the cloaca into the caudal vein and collecting the blood (<1% of body volume) into heparinized syringes. Animals were then euthanized by severing the spinal cord using the method described by Speers-Roesch *et al.* (2012b) so that tissues could be sampled. Whole blood [Hb] was determined using the HemoCue^®^ (Hb 201 System, Australia Pty Ltd) with 10 µl of whole blood and was reported as grams per 100 ml using a calibration curve according to Clark *et al.* (2008) corrected for tropical reef species by Rummer *et al.* (2013b). The Hct was determined by centrifuging 60 µl of whole blood in heparinized microcapillary tubes for 3 min at 17 000***g*** and calculated as the ratio of packed red blood cells to total blood volume (as a percentage). Both [Hb] and Hct were used to calculate the MCHC. The spleen was dissected from each shark and weighed to the nearest 0.001 g. The SSI was calculated as the ratio of the spleen to body mass (as a percentage). Plasma was flash frozen immediately in liquid nitrogen and then stored at −80°C until analysis for [HCO_3_^−^] via colorometrically linked enzyme assay and for [Na^+^], [K^+^], [Cl^−^] (1:1 dilution with deionized water) and [urea] (1:19 dilution with deionized water) via ion-specific electrodes (ISE; Beckman Coulter System AU480). Heart, brain and red muscle samples were also collected and frozen in liquid N_2_ for citrate synthase enzyme analysis according to McClelland *et al.* (2005). Briefly, frozen tissues were homogenized in a standard buffer solution containing 5 mm EDTA, 0.1% Triton X-100, 0.2 mm dithiothreitol and 50 mm Hepes (adjusted to pH 7.4) and stored at −80°C. The citrate synthase assay buffer contained (mm): 20 Tris (pH 8.0), 0.1 5,5-dithiobis (2-nitrobenzoic acid) and 0.3 acetyl-CoA. The reaction was initiated by the addition of 0.5 mm oxaloacetate, and absorbance was measured for 5 min at 412 nm. Control samples were assayed without oxaloacetate to control for background hydrolase activity.

#### Calculations and statistical analyses

Raw text files created for the Firesting recordings were imported offline into LabChart version 6.1.3 (ADInstruments, Colorado Springs, CO, USA), which was used to analyse data. A modified version of equations from Bushnell *et al.* (1994) and Schurmann and Steffensen (1997) was used to calculate *Ṁ*O_2Rest_ (in milligams per kilogram per hour). To do this, the average of the shallowest 10% of slopes [change in O_2_ concentration over a period of 15–20 min (in milligams of O_2_ per litre per second) in between flushing cycles] was determined for each individual shark. From this, the appropriate proportion of background O_2_ consumption, which was measured 2–3 h before and after each trial for each respirometer and assumed linear, was subtracted. This value was then multiplied by the volume of the respirometer (in litres; minus the volume of the fish), all of which was divided by the mass of the fish (in kilograms). Respirometers were cleaned daily to ensure that background (microbial) respiration did not exceed 10% of the *Ṁ*O_2Rest_ of the sharks. Means and SEM for *Ṁ*O_2Rest_ were calculated for each of the three CO_2_ treatments.

**Figure 1:**
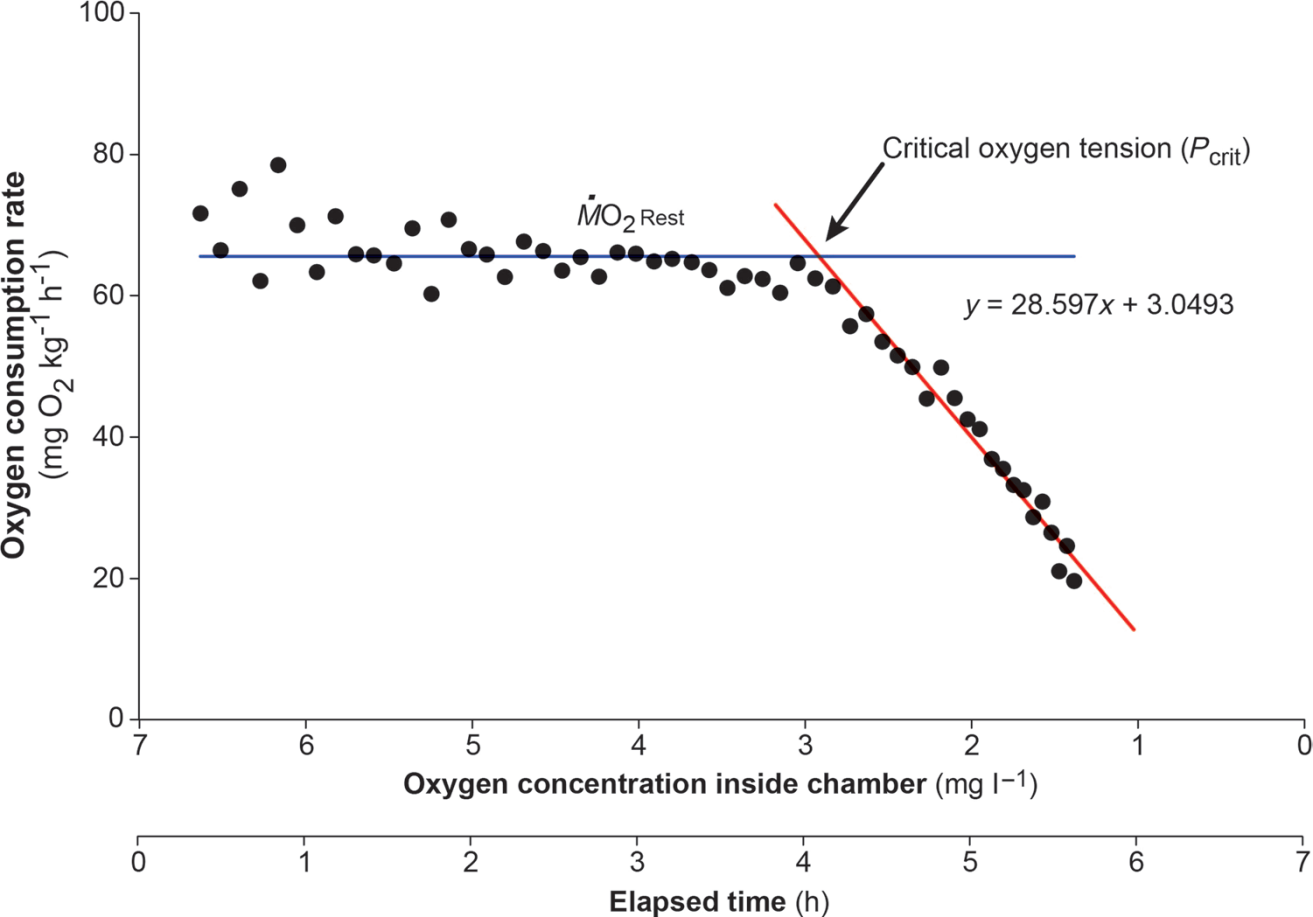
Representative trace illustrating the changes in oxygen consumption rate of an individual epaulette shark (*Hemiscyllium ocellatum*) as the oxygen concentration of the water decreased and the time over which this occurred. The parallel line represents the resting oxygen consumption rate. After the respirometry chamber was sealed, the oxygen consumption rate began to decrease below resting levels. The diagonal line is a trend line, with the intersection of both lines demarcating the critical oxygen tension (*P*_crit_).

A similar data extraction and calculation protocol was followed for determining *P*_crit_. Again, *Ṁ*O_2Rest_ was calculated for each shark from the shallowest 10% of slopes that were recorded prior to sealing the respirometer. Then, the mean slope for every 5 min period of time while the respirometer was sealed was extracted (usually 20–30 slopes), and *Ṁ*O_2_ values were calculated from those slopes. To determine *P*_crit_, all *Ṁ*O_2_ values were plotted against the oxygen concentration within the chamber for each shark. A horizontal (regression) line was fitted to the mean *Ṁ*O_2Rest_ prior to sealing the respirometer. Then, a linear regression was applied to all of the points that consecutively fell below *Ṁ*O_2Rest_ once the respirometer had been sealed. The point at which both regression lines intersected was reported as the critical oxygen tension or *P*_crit_ (in milligams of O_2_ per litre) for that individual (Fig. 1; Ott *et al.*, 1980; Nilsson *et al.*, 2004; Collins *et al.*, 2013). Means and SEM for *P*_crit_ were calculated for each CO_2_ treatment.

Nested ANOVA, with holding tanks nested within CO_2_ treatments, was first used to test whether there was a significant effect of holding tank on mean *Ṁ*O_2Rest_ (in milligams per kilogram per hour) or mean *P*_crit_ (in milligams of O_2_ per litre). As there was no significant effect of tanks on either parameter, data from the two tanks within treatments were pooled for further analyses. ANCOVA was used to compare *Ṁ*O_2Rest_ among the three CO_2_ treatments, with standard length as a covariate. To compare *P*_crit_ among treatment groups, a robust regression analysis was performed with standard length as a covariate. Robust regression analysis was chosen over ANCOVA for *P*_crit_ analysis due to potential outliers that could otherwise be solely responsible for significant outcomes. The removal of such outliers was rejected owing to the relatively small sample size. Instead, robust regression weighs values differently based on their chance of being an outlier. Hence, the further away a single data point was from the mean, the less influential it became for the statistical outcome of the analysis. There was no interaction between the main effect (CO_2_) and the covariate in either analysis; therefore, to increase statistical power, the analyses were run again without this term included. Standard length was not included in haematological and tissue data analyses because it had no significant effect on the outcomes. ANOVAs were then used together with Holm–Sidak *post hoc* tests to compare haematological and tissue parameters between animals acclimated to control, medium and high CO_2_ conditions. Statistical significance was accepted when *P* < 0.05. All analyses were carried out using S-Plus (TIBCO Software Inc., Palo Alto, CA, USA).

## Results

### Resting oxygen consumption rates

**Figure 2:**
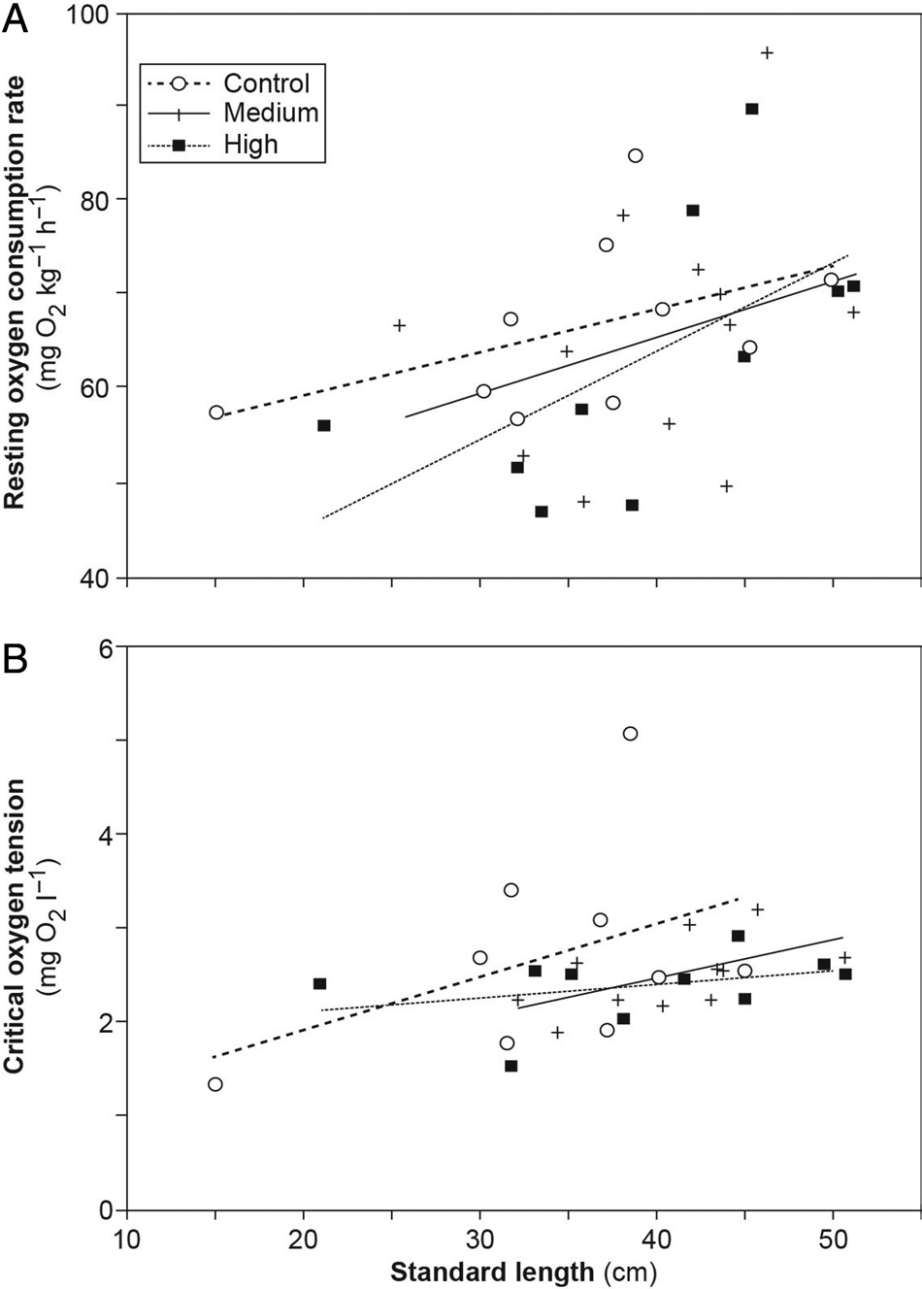
The relationship between resting oxygen consumption rate (**A**) and critical oxygen tension (*P*_crit_; **B**) with the standard length of individual sharks from control, medium and high CO_2_ treatment groups. Please refer to Materials and methods for further details regarding calculations.

There were no significant differences in *Ṁ*O_2Rest_ values between CO_2_ treatment groups (*F*_2,28_ = 0.578; *P* = 0.568). However, *Ṁ*O_2Rest_ depended on the standard length of the individuals, with larger animals having a higher *Ṁ*O_2Rest_ than smaller animals (*F*_2,28_ = 6.70; *P* = 0.0151; Fig. 2A). Values for *Ṁ*O_2Rest_ ranged from 46.8 to 95.4 mg O_2_ kg^−1^ h^−1^ with a mean of 65.2 ± 2.13 mg O_2_ kg^−1^ h ^−1^ across all treatments.

### Critical oxygen tension

The *P*_crit_ values did not differ significantly between CO_2_ treatment groups (*t*_4,26_ = − 0.170; *P* = 0.866). However, standard length had a significant effect on the *P*_crit_ of individuals (*t*_4,26_ = 2.26; *P* = 0.0323; Fig. 2B), with larger animals reaching *P*_crit_ at a higher seawater O_2_ concentration than smaller animals. The *P*_crit_ values ranged from 1.32 to 5.07 mg O_2_ l^−1^ with a mean of 2.51 ± 0.122 mg O_2_ l^−1^ across all treatment groups.

### Haematology and tissue samples

**Figure 3:**
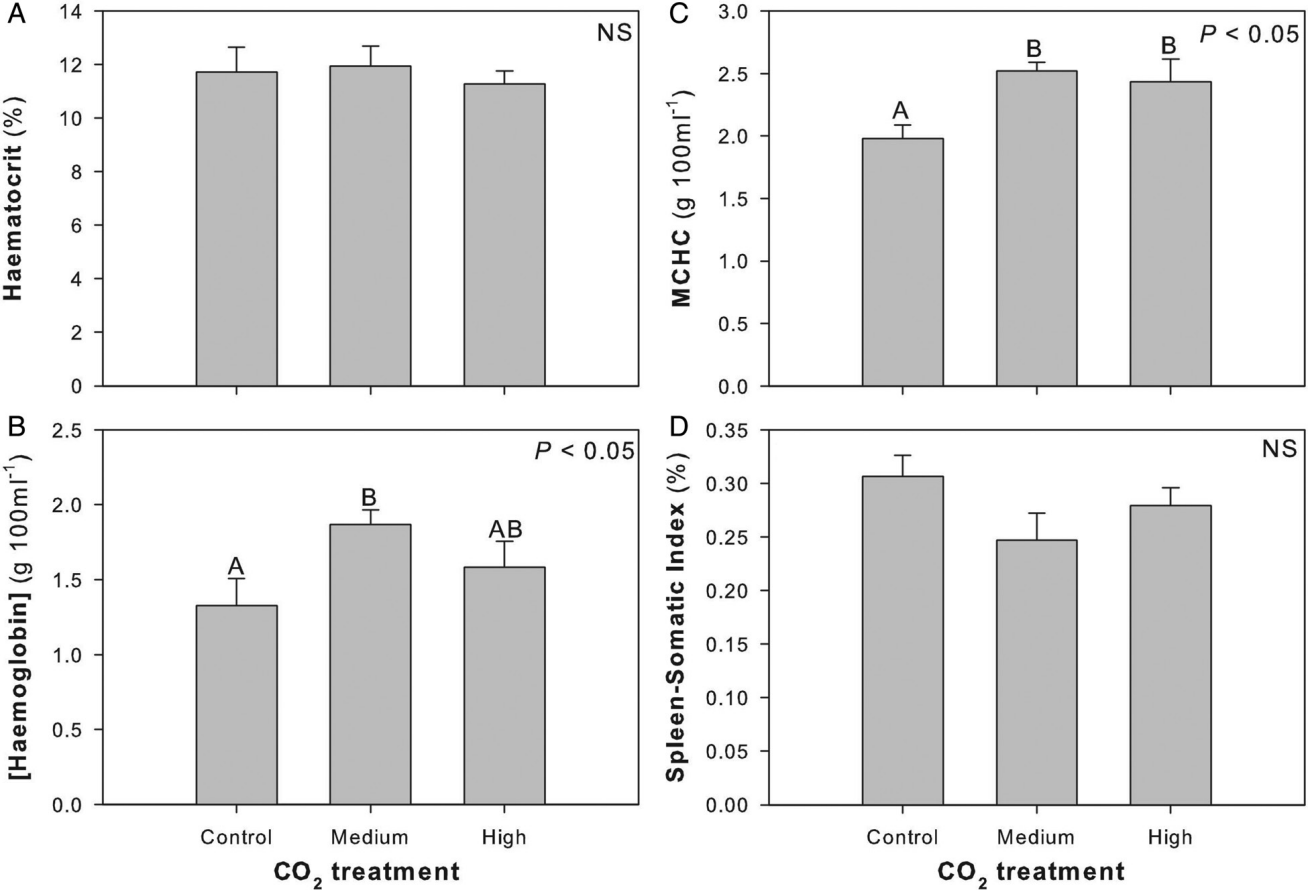
Changes in haematocrit (**A**), haemoglobin concentration (**B**), mean cell haemoglobin concentration (MCHC; **C**), and spleen–somatic index (**D**) after sharks were exposed to control, medium or high CO_2_ for ∼90 days. Different letters within a panel demarcate significant differences between treatment groups, and statistical significance is noted in the top right corner of each panel. Abbreviation: NS, not significant.

**Figure 4:**
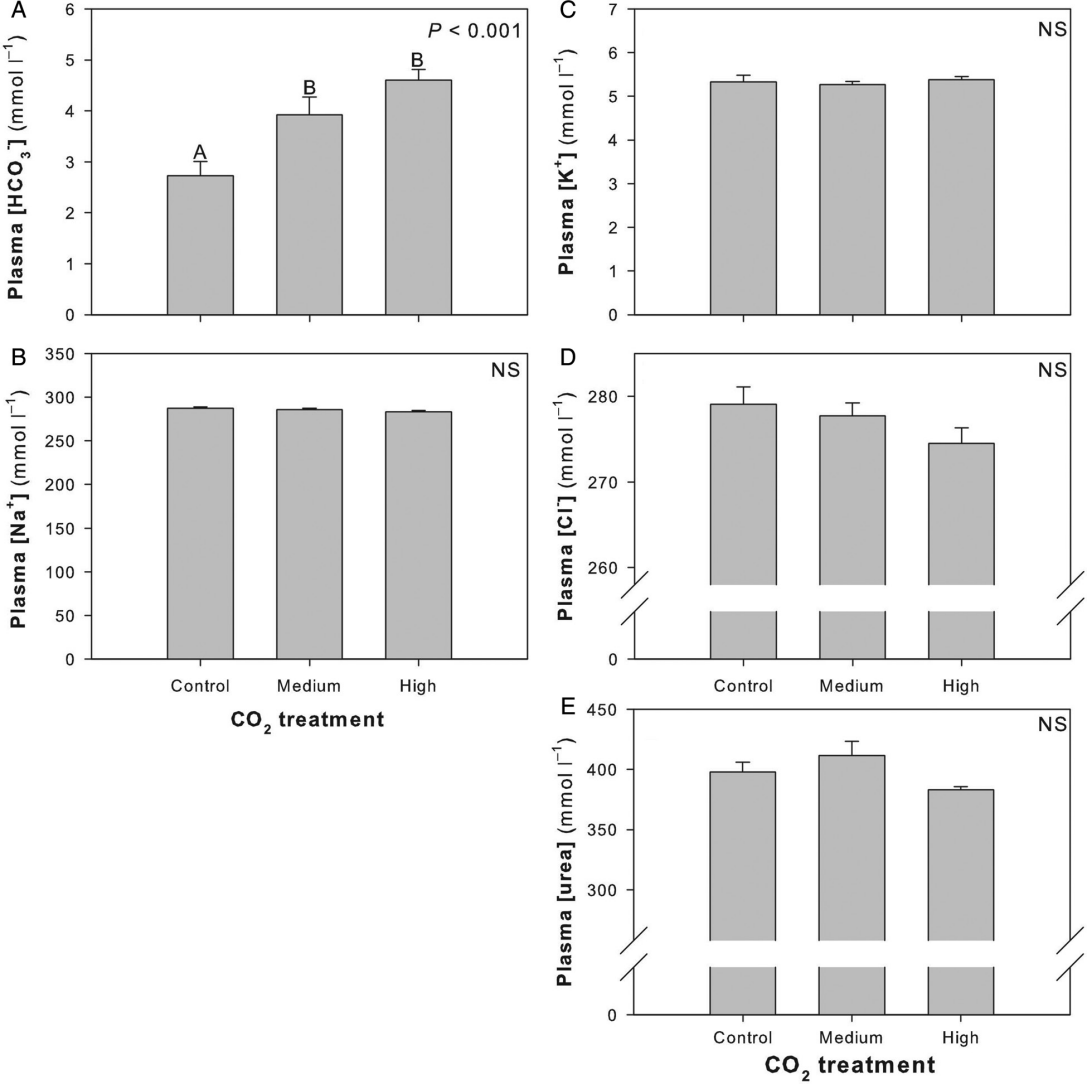
Changes in plasma parameters after sharks were exposed to control, medium or high CO_2_ for ∼90 days. Different letters within a panel demarcate significant differences between treatment groups, and statistical significance is noted in the top right corner of each panel. Abbreviation: NS, not significant.

**Figure 5:**
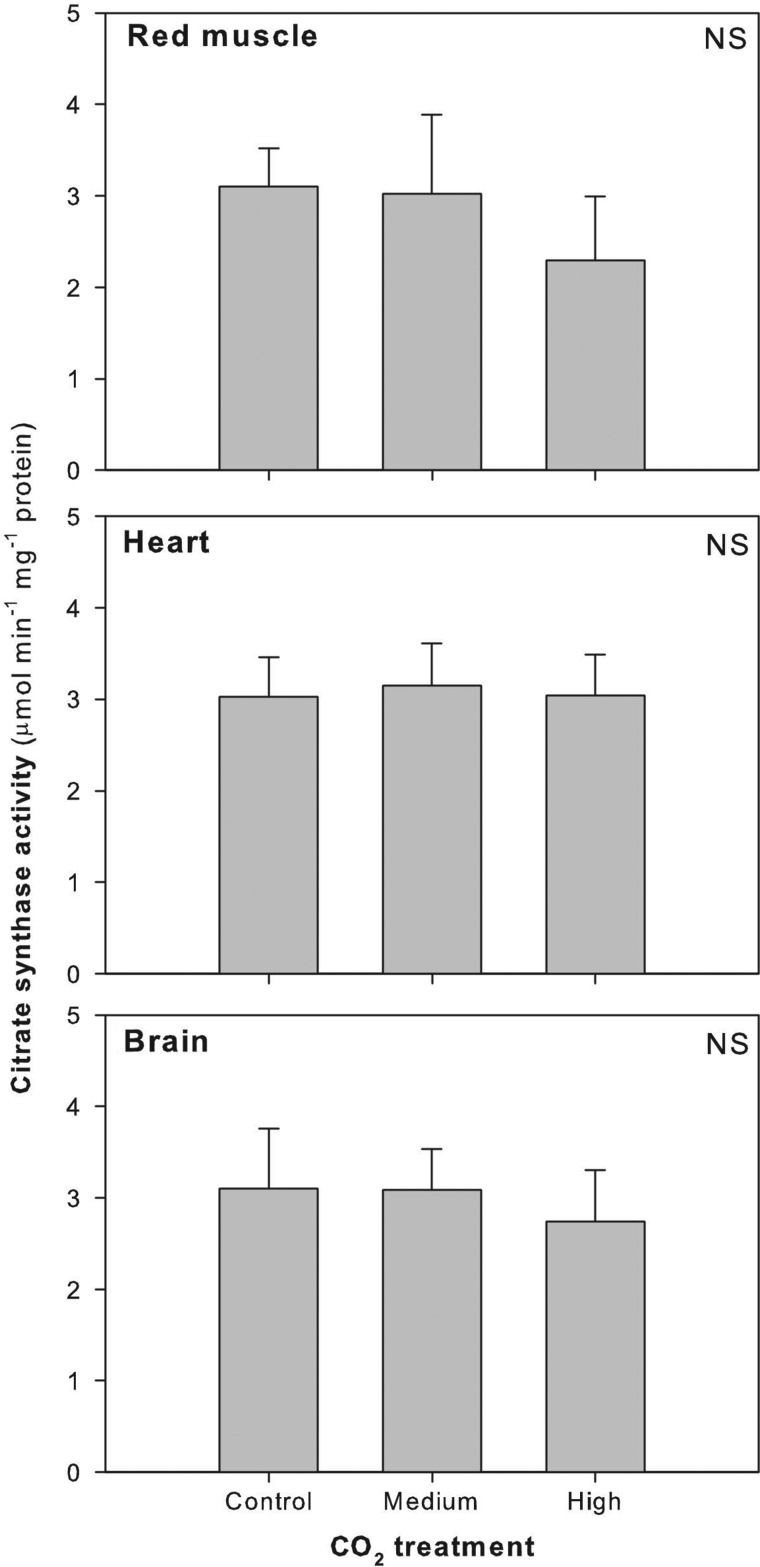
Changes in red muscle, heart and brain citrate synthase enzyme activity after sharks were exposed to control, medium or high CO_2_ for ∼90 days. Statistical significance is noted in the top right corner of each panel. Abbreviation: NS, not significant.

No significant differences were detected in Hct between CO_2_ treatment groups (*F*_2,22_ = 0.214; *P* = 0.809; Fig. 3A). There was a significant increase in [Hb] between the control and the medium CO_2_ treatment groups (*F*_2,23_ = 3.447; *P* = 0.048; Fig. 3B), an elevation that was maintained with the high CO_2_ treatment group for MCHC values (*F*_2,21_ = 5.067; *P* = 0.0160; Fig. 3C). Although not significant, there was a trend toward decreased SSI with high CO_2_ exposure (*F*_2,22_ = 2.050; *P* = 0.153; Fig. 3D). There was a significant increase in plasma [HCO_3_^−^] in both the medium and high CO_2_ treatment groups (*F*_2,21_ = 10.893; *P* < 0.001; Fig. 4A). However, there was no difference in plasma [Na^+^], [K^+^], [Cl^−^] or [urea] between control and CO_2_ treatment groups ([Na^+^], *F*_2,21_ = 1.543, *P* = 0.237, Fig. 4B; [K^+^], *F*_2,21_ = 0.247, *P* = 0.783, Fig. 4C; [Cl^−^], *F*_2,21_ = 1.697, *P* = 0.207, Fig. 4D; and [urea], *F*_2,21_ = 2.907, *P* = 0.077, Fig. 4E). Citrate synthase activity did not change significantly between control and CO_2_ treatment groups in red muscle (*F*_2,16_ = 0.371; *P* = 0.696), heart (*F*_2,18_ = 0.0238; *P* = 0.976) or brain (*F*_2,19_ = 0.131; *P* = 0.878; Fig. 5).

## Discussion

Long-term exposure to near-future CO_2_ conditions did not significantly affect metabolic performance or hypoxia sensitivity of epaulette sharks. In contrast, changes in [Hb] and MCHC were evident after ∼90 days of exposure to 600 µatm CO_2_ levels, and plasma [HCO_3_^−^] was elevated in both the moderate and high CO_2_ treatment groups, suggesting that physiological adjustments were being made to cope with elevated CO_2_ at the level of oxygen transport and ion regulation. However, there was no increase in metabolic capacity at the level of the mitochondria, as indicated by the lack of change in citrate synthase activity. Our findings suggest that, for this reef-inhabiting benthic elasmobranch, neither the energetic costs of basic maintenance nor sensitivity to hypoxia may be compromised in the elevated CO_2_ conditions projected for the end of this century.

The compensatory mechanisms used by *H. ocellatum* to maintain resting metabolic rates in normoxic and hypoxic conditions after prolonged exposure to elevated CO_2_ may be linked to maintaining oxygen uptake and delivery and ion regulation. Following ∼90 days of CO_2_ exposure, epaulette sharks exhibited a significant increase in [Hb] and MCHC. Short-term changes in haematological parameters have been documented in teleosts and elasmobranchs following capture, cannulation and exercise (Soivio *et al.*, 1973; Wood *et al.*, 1977; Turner *et al.*, 1983; Wells *et al.*, 1986), upon acclimation to elevated temperature (adult horn sharks, *Heterodontus francisci*; Neale *et al.*, 1977) and in response to anoxia (grey carpet shark, *Chiloscyllium punctatum*, and epaulette shark; Chapman and Renshaw, 2009). In teleosts, acute changes can be associated with adrenergic red blood cell (RBC) swelling (Caldwell *et al.*, 2006), a mechanism in place to protect RBC pH and oxygen transport during stress, but not known to occur in elasmobranchs (Berenbrink *et al.*, 2005). Both teleosts and elasmobranchs do, however, use their spleen to produce and store RBCs (Turner *et al.*, 1983; Fänge and Nilsson, 1985; Lai *et al.*, 2006) and can contract it to increase the proportion of RBCs in the circulation (Ken-Ichi, 1988; Lai *et al.*, 2006), presumably to aid in oxygen transport (Jensen *et al.*, 1992). We observed a decrease, although non-significant, in the SSI in sharks exposed to both medium and high CO_2_, suggesting that splenic contraction(s) may have occurred at some point during the CO_2_ exposure period. Periodic splenic contractions could also increase the proportion of immature RBCs in circulation, which could explain the slight increase in MCHC without significant changes in Hct. The temporal scale of splenic RBC release and subsequent increases in erythropoietin, the glycoprotein responsible for regulating RBC numbers, is well understood for teleosts (Lai *et al.*, 2006) and could be similar in elasmobranchs exposed to elevated CO_2_ over extended periods of time, which is worth further investigation.

Plasma [HCO_3_^−^] was elevated in sharks upon 90 days of exposure to elevated CO_2_, which indicates some level of long-term acid–base compensation. This finding is supported by studies by Deigweiher *et al.* (2008), in which acclimation to elevated CO_2_ over 6 weeks in a marine teleost resulted in upregulation of Na^+^/HCO_3_^−^ cotransporters (NBC1) and Na^+^–K^+^-ATPase at higher densities. Given the relationship between bicarbonate availability and synthesis of urea (the predominant osmolyte used by most elasmobranchs), acid–base compensatory mechanisms could have affected [urea] and therefore the efficiency of osmoregulatory pathways (Wood *et al.*, 1995). As [urea] did not change with CO_2_ exposure, this may not be problematic at the CO_2_ levels used here and/or over the 90 day duration. The activity of citrate synthase, the first enzyme of the Krebs cycle located within the mitochondria, can be a good indicator of aerobic capacity. Unchanged citrate synthase activity after prolonged CO_2_ exposure further suggests that there is no limitation at the level of aerobic energy production in any of the tested tissues (McClelland *et al.*, 2005). Although there may have been no changes to aerobic capacity, changes may have been occurring in anaerobic pathways (e.g. activity of lactate dehydrogenase, the last enzyme of anaerobic glycolysis) to maintain energy production. This would be worthy of further investigation. As Esbaugh *et al.* (2012) suggest, species that are already adapted to low levels of CO_2_ may no longer rely on traditional short-term acid–base compensation strategies but instead use morphological changes (e.g. gill permeability, diffusion distances) or alter chemical equilibrium constraints in the blood over longer periods to maintain oxygen transport.

While there were no changes in metabolic performance in the sharks upon long-term CO_2_ exposure, there was an unexpected pattern of mass-specific metabolic rates, with larger sharks exhibiting higher mass-specific metabolic rates than smaller sharks. This contradicts the usual pattern exhibited by ectotherms, but may be related to their feeding patterns. For example, we examined sharks ranging in size from ∼20 to 50 cm. However, we used a set 48 h fasting period prior to determining oxygen consumption rates and prior to blood and tissue sampling because of their small size and benthic lifestyle and previous feeding patterns while in captivity. It could have been that 48 h was sufficient fasting time for the smaller animals but not for the larger animals of that size range (Wood *et al.*, 2007). Therefore, the larger animals could have been exhibiting slightly increased metabolic rates due to specific dynamic action, which could also mask any acid–base processes occurring due to CO_2_ exposure. The relationship between acid–base disturbances originating from feeding and those due to elevated water CO_2_ has ecological relevance and should be investigated in future studies.

Environment and lifestyle play an important role in physiological tolerance to changing environmental conditions (Pörtner and Farrell, 2008), and this study confirms that *H. ocellatum* is no exception. It is already known that *H. ocellatum* exhibits the lowest value of *P*_crit_ shown for any elasmobranch tested to date, suggesting an exceptional tolerance to short-term hypoxia, which is unique among chondrichthyans (Wise *et al.*, 1998; Routley *et al.*, 2002). *Hemiscyllium ocellatum* occupies shallow reef platforms that are subject to dramatic diurnal fluctuations in environmental O_2_ and CO_2_ conditions (Routley *et al.*, 2002; Diaz and Breitburg, 2009; Last and Stevens, 2009). During calm nights, the low O_2_ tension encountered on coral reefs can drop below 10% air saturation (Routley *et al.*, 2002), usually as a result of respiration by reef organisms and especially during nocturnal low tides. This can also result in elevations in PCO_2_, which have been reported to exceed 1000 µatm on shallow reef flats at night (Ohde and van Woesik, 1999; Shaw *et al.*, 2013). The CO_2_ levels may even be higher in caves, reef crevices and restricted-flow habitats, which are used by *H. ocellatum* for shelter (Compagno, 2002; Last and Stevens, 2009). Indeed, diurnal or acute fluctuations in O_2_ and CO_2_ may play a role in signalling metabolism in species using such habitats. However, acute responses often differ dramatically from responses to prolonged exposure, and it is important to make this distinction. The increased uptake of CO_2_ by the ocean will affect both the average CO_2_ level and the magnitude of extreme CO_2_ fluctuations (Ohde and van Woesik, 1999; Shaw *et al.*, 2013). This makes our finding that *H. ocellatum* exhibited no change in metabolic performance, including sensitivity to hypoxia, after prolonged exposure to projected future CO_2_ levels even more important.

Adaptation to life on shallow reef platforms and lagoons may be the key to species like *H. ocellatum* for maintaining performance in projected future CO_2_ concentrations (Melzner *et al.*, 2009b). While noteworthy, what was previously known about the physiological tolerance of the epaulette shark to challenging environmental conditions was related to acute exposure of minutes to hours. This is extremely relevant to a shelter-seeking, benthic, reef-dwelling species like the epaulette shark that would experience such conditions burrowing into coral caves to avoid predation or to exploit food sources, activities vital to biological fitness. Pelagic shark species, many of which function as apex predators in their respective environments (Last and Stevens, 2009), however, do not typically exhibit shelter-seeking behaviours in areas that would experience the routine fluctuations in water chemistry experienced by *H. ocellatum* and therefore may not tolerate prolonged periods of elevated CO_2_. Given that increased uptake of CO_2_ by the ocean may mean that the high CO_2_ levels that the epaulette shark may already routinely experience could be the new average ocean CO_2_ levels, some species may be able to tolerate future conditions better. Future studies should investigate the importance of fluctuating environmental conditions in shaping an organism's tolerance. Differential effects on functional groups could impact predator–prey dynamics, affect the population structure of elasmobranchs and other aquatic organisms inhabiting coral reefs and, ultimately, impact ecosystem health. Investigating both sensitive and tolerant species from an array of habitat types would help to tease apart the role of the environment from other factors, including evolutionary history and behaviour, all of which is important when considering conservation measures under future climate change scenarios.

## Funding

This work was supported by funding from the School of Marine and Tropical Biology (D.D.U.H., P.L.M.); the School of Earth and Environmental Science (C.A.S., M.R.H.); AIMS@JCU (D.D.U.H., M.R.H.); and the Australian Research Council Centre of Excellence for Coral Reef Studies (J.L.R., P.L.M.).

## References

[COU047C1] Berenbrink M Koldkjær P Kepp O Cossins AR (2005) Evolution of oxygen secretion in fishes and the emergence of a complex physiological system. Science 307: 1752–1757. 15774753 10.1126/science.1107793

[COU047C2] Brauner CJ Baker DW (2009) Patterns of acid-base regulation during exposure to hypercarbia in fishes. In Glass ML Wood SC, eds, Cardio-Respiratory Control in Vertebrates. Springer, Heidelberg, pp 43–63.

[COU047C3] Bushnell P Steffensen J Schurmann H Jones D (1994) Exercise metabolism in two species of cod in arctic waters. Polar Biol 14: 43–48.

[COU047C4] Caldwell S Rummer JL Brauner CJ (2006) Blood sampling techniques and storage duration: effects on the presence and magnitude of the red blood cell β-adrenergic response in rainbow trout (*Oncorhynchus mykiss*). Comp Biochem Physiol A Mol Integr Physiol 144: 188–195. 16713317 10.1016/j.cbpa.2006.02.029

[COU047C5] Chapman CA Renshaw G (2009) Hematological responses of the grey carpet shark (*Chiloscyllium punctatum*) and the epaulette shark (*Hemiscyllium ocellatum*) to anoxia and re–oxygenation. J Exp Zool A Ecol Genet Physiol 311: 422–438. 19405134 10.1002/jez.539

[COU047C6] Claiborne JB Evans DH (1992) Acid-base balance and ion transfers in the spiny dogfish (*Squalus acanthias*) during hypercapnia: a role for ammonia excretion. J Exp Zool 261: 9–17.

[COU047C7] Clark TD Eliason EJ Sandblom E Hinch SG Farrell AP (2008) Calibration of a hand-held haemoglobin analyser for use on fish blood. J Fish Biol 73: 2587–2595.

[COU047C8] Collins GM Clark TD Rummer JL Carton AG (2013) Hypoxia tolerance is conserved across genetically distinct sub-populations of an iconic, tropical Australian teleost (*Lates calcarifer*). Conserv Physiol 1: doi:10.1093/conphys/cot029. 10.1093/conphys/cot029 PMC4806625 27293613

[COU047C9] Compagno LJ (2002) Sharks of the World: An Annotated and Illustrated Catalogue of Shark Species Known to Date. Vol. 2, Bullhead, Mackeral and Carpet Sharks (Heterodontiformes, Lamniformes and Orecto-lobiformes) Rome, Food and Agriculture Organization of the United Nations

[COU047C10] Couturier CS Stecyk JAW Rummer JL Munday PL Nilsson GE (2013) Species-specific effects of near-future CO_2_ on the respiratory performance of two tropical prey fish and their predator. Comp Biochem Physiol A Mol Integr Physiol 166: 482–489. 23916817 10.1016/j.cbpa.2013.07.025 PMC3830952

[COU047C11] Deigweiher K Koschnick N Pörtner H-O Lucassen M (2008) Acclimation of ion regulatory capacities in gills of marine fish under environmental hypercapnia. Am J Physiol Regul Integr Comp Physiol 295: R1660–R1670. 18799636 10.1152/ajpregu.90403.2008

[COU047C12] Diaz RJ Breitburg DL (2009) The hypoxic environment. Fish Physiol 27: 1–23.

[COU047C13] Dickson A Millero F (1987) A comparison of the equilibrium constants for the dissociation of carbonic acid in seawater media. Deep Sea Res 34: 1733–1743.

[COU047C14] Doney SC Schimel DS (2007) Carbon and climate system coupling on timescales from the Precambrian to the Anthropocene. Ann Rev Environ Resour 32: 31–66.

[COU047C15] Doney SC Fabry VJ Feely RA Kleypas JA (2009) Ocean acidification: the other CO_2_ problem. Annu Rev Mar Sci 1: 169–192. 10.1146/annurev.marine.010908.163834 21141034

[COU047C16] Dowd WW Renshaw GM Cech JJ Jr Kultz D (2010) Compensatory proteome adjustments imply tissue-specific structural and metabolic reorganization following episodic hypoxia or anoxia in the epaulette shark (*Hemiscyllium ocellatum*). Physiol Genomics 42: 93–114. 20371547 10.1152/physiolgenomics.00176.2009 PMC2888556

[COU047C17] Esbaugh AJ Heuer R Grosell M (2012) Impacts of ocean acidification on respiratory gas exchange and acid–base balance in a marine teleost, *Opsanus beta*. J Comp Physiol B 182: 921–934. 22581071 10.1007/s00360-012-0668-5

[COU047C18] Evans DH (1982) Mechanisms of acid extrusion by two marine fishes: the teleost, *Opsanus beta*, and the elasmobranch, *Squalus acanthias*. J Exp Biol 97: 289–299.

[COU047C19] Fabry VJ Seibel BA Feely RA Orr JC (2008) Impacts of ocean acidification on marine fauna and ecosystem processes. ICES J Mar Sci 65: 414–432.

[COU047C20] Fänge R Nilsson S (1985) The fish spleen: structure and function. Experientia 41: 152–158. 3972063 10.1007/BF02002607

[COU047C21] Henriksson P Mandic M Richards JG (2008) The osmorespiratory compromise in sculpins: impaired gas exchange is associated with freshwater tolerance. Physiol Biochem Zool 81: 310–319. 18419557 10.1086/587092

[COU047C22] Hoegh-Guldberg O Mumby PJ Hooten AJ Steneck RS Greenfield P Gomez E Harvell CD Sale PF Edwards AJ Caldeira K (2007) Coral reefs under rapid climate change and ocean acidification. Science 318: 1737–1742. 18079392 10.1126/science.1152509

[COU047C23] IPCC (2013) Climate Change 2013: The Physical Science Basis. Contribution of Working Group I to the Fifth Assessment Report of the Intergovernmental Panel on Climate Change. Stocker TF Qin D Plattner G-K Tignor M Allen SK Boschung J Nauels A Xia Y Bex V Midgley PM, eds. Cambridge, UK and New York, NY, USA, Cambridge University Press.

[COU047C24] Ishimatsu A Hayashi M Kikkawa T (2008) Fishes in high-CO_2_, acidified oceans. Mar Ecol Prog Ser 373: 295–302.

[COU047C25] Jensen FB Nikinmaa M Weber RE (1993) Environmental perturbations of oxygen transport in teleost fishes: causes, consequences and compensations. In Rankin JC Jensen F, eds, Fish Ecophysiology. Springer, The Netherlands, pp 161–179.

[COU047C26] Ken-Ichi Y (1988) Contraction of spleen in exercised freshwater teleost. Comp Biochem Physiol A Physiol 89: 65–66.

[COU047C27] King PA Goldstein L (1983) Renal ammoniagenesis and acid excretion in the dogfish, *Squalus acanthias*. Am J Physiol Regul Integr Comp Physiol 245: R581–R589. 10.1152/ajpregu.1983.245.4.R581 6137960

[COU047C28] Lai JC Kakuta I Mok HO Rummer JL Randall DJ (2006) Effects of moderate and substantial hypoxia on erythropoietin levels in rainbow trout kidney and spleen. J Exp Biol 209: 2734–2738. 16809464 10.1242/jeb.02279

[COU047C29] Last PR Stevens JD (2009) Sharks and Rays of Australia. Cambridge, MA, USA, Harvard University Press.

[COU047C30] Lüthi D Le Floch M Bereiter B Blunier T Barnola J-M Siegenthaler U Raynaud D Jouzel J Fischer H Kawamura K (2008) High-resolution carbon dioxide concentration record 650,000–800,000 years before present. Nature 453: 379–382. 18480821 10.1038/nature06949

[COU047C31] Marais E Chown SL (2008) Beneficial acclimation and the Bogert effect. Ecol Lett 11: 1027–1036. 18616546 10.1111/j.1461-0248.2008.01213.x

[COU047C32] McClelland GB Dalziel AC Fragoso NM Moyes CD (2005) Muscle remodeling in relation to blood supply: implications for seasonal changes in mitochondrial enzymes. J Exp Biol 208: 515–522. 15671340 10.1242/jeb.01423

[COU047C33] Melzner F Göbel S Langenbuch M Gutowska MA Pörtner H-O Lucassen M (2009a) Swimming performance in Atlantic cod (*Gadus morhua*) following long-term (4–12 months) acclimation to elevated seawater PCO_2_. Aquat Toxicol 92: 30–37. 19223084 10.1016/j.aquatox.2008.12.011

[COU047C34] Melzner F Gutowska M Langenbuch M Dupont S Lucassen M Thorndyke MC Bleich M Pörtner HO (2009b) Physiological basis for high CO_2_ tolerance in marine ectothermic animals: pre-adaptation through lifestyle and ontogeny? Biogeosciences 6: 2313–2331.

[COU047C35] Munday PL Crawley NE Nilsson GE (2009) Interacting effects of elevated temperature and ocean acidification on the aerobic performance of coral reef fishes. Mar Ecol Prog Ser 388: 235–242.

[COU047C36] Neale NL Honn KV Chavin W (1977) Hematological responses to thermal acclimation in a cold water squali-form (*Heterodontus francisci* Girard 1984). J Comp Physiol 115: 215–222.

[COU047C37] Nilsson GE Renshaw GM (2004) Hypoxic survival strategies in two fishes: extreme anoxia tolerance in the North European crucian carp and natural hypoxic preconditioning in a coral-reef shark. J Exp Biol 207: 3131–3139. 15299034 10.1242/jeb.00979

[COU047C38] Nilsson GE Hobbs J-P Munday PL Östlund-Nilsson S (2004) Coward or braveheart: extreme habitat fidelity through hypoxia tolerance in a coral-dwelling goby. J Exp Biol 207: 33–39. 14638830 10.1242/jeb.00713

[COU047C39] Ohde S van Woesik R (1999) Carbon dioxide flux and metabolic processes of a coral reef, Okinawa. Bull Mar Sci 65: 559–576.

[COU047C40] Orr JC Fabry VJ Aumont O Bopp L Doney SC Feely RA Gnanadesikan A Gruber N Ishida A Joos F (2005) Anthropogenic ocean acidification over the twenty-first century and its impact on calcifying organisms. Nature 437: 681–686. 16193043 10.1038/nature04095

[COU047C41] Ott ME Heisler N Ultsch GR (1980) A re-evaluation of the relationship between temperature and the critical oxygen tension in freshwater fishes. Comp Biochem Physiol A Physiol 67: 337–340.

[COU047C42] Pierrot D Lewis E Wallace DWR (2006) MS Excel Program Developed for CO_2_ System Calculations. Carbon Dioxide Information Analysis Center, Oak Ridge National Laboratory, US Department of Energy, Oak Ridge, TN, USA. http://cdiac.ornl.gov/ftp/co2sys/CO2SYS_calc_XLS_v2.1/:

[COU047C43] Pörtner HO (2008) Ecosystem effects of ocean acidification in times of ocean warming: a physiologist's view. Mar Ecol Prog Ser 373: 203–217.

[COU047C44] Pörtner HO Farrell AP (2008) Physiology and climate change. Science 322: 690–692. 18974339 10.1126/science.1163156

[COU047C45] Raven J Caldeira K Elderfield H Hoegh-Guldberg O Liss P Riebesell U Shepherd J Turley C Watson A (2005) Ocean Acidification Due to Increasing Atmospheric Carbon Dioxide. The Royal Society, London, UK.

[COU047C46] Renshaw GMC Kerrisk CB Nilsson GE (2002) The role of adenosine in the anoxic survival of the epaulette shark, *Hemiscyllium ocellatum*. Comp Biochem Physiol B Biochem Mol Biol 131: 133–141. 11818236 10.1016/s1096-4959(01)00484-5

[COU047C47] Rosa R Seibel BA (2008) Synergistic effects of climate-related variables suggest future physiological impairment in a top oceanic predator. Proc Natl Acad Sci USA 105: 20776–20780. 19075232 10.1073/pnas.0806886105 PMC2634909

[COU047C48] Routley MH Nilsson GE Renshaw GMC (2002) Exposure to hypoxia primes the respiratory and metabolic responses of the epaulette shark to progressive hypoxia. Comp Biochem Physiol A Mol Integr Physiol 131: 313–321. 11818221 10.1016/s1095-6433(01)00484-6

[COU047C49] Rummer JL McKenzie DJ Innocenti A Supuran CT Brauner CJ (2013a) Enhanced muscle oxygen delivery may represent the incipient function of the Root effect in ray-finned fishes. Science 340: 1327–1329. 23766325 10.1126/science.1233692

[COU047C50] Rummer JL Stecyk JAW Couturier CS Watson S-A Nilsson GE Munday PL (2013b) Elevated CO_2_ enhances aerobic scope of a coral reef fish. Conserv Physiol 1: doi:10.1093/conphys/cot023. 10.1093/conphys/cot023 PMC4732439 27293607

[COU047C51] Sabine CL Feely RA (2007) The oceanic sink for carbon dioxide. Chapter 3. In Reay D Hewitt N Grace J Smith K, eds, Greenhouse Gas Sinks, CABI Publishing, Oxfordshire, UK, pp 31–49.

[COU047C52] Sabine CL Feely RA Gruber N Key RM Lee K Bullister JL Wanninkhof R Wong CS Wallace DWR Til-brook B (2004) The oceanic sink for anthropogenic CO_2_. Science 305: 367–371. 15256665 10.1126/science.1097403

[COU047C53] Schurmann H Steffensen JF (1997) Effects of temperature, hypoxia and activity on the metabolism of juvenile Atlantic cod. J Fish Biol 50: 1166–1180.

[COU047C54] Shaw EC McNeil BI Tilbrook B Matear R Bates ML (2013) Anthropogenic changes to seawater buffer capacity combined with natural reef metabolism induce extreme future coral reef CO_2_ conditions. Glob Change Biol 19: 1632–1641. 10.1111/gcb.12154 23505026

[COU047C55] Soivio A Nyholm K Westman K (1973) Notes on haematocrit determinations on rainbow trout, *Salmo gairdneri*. Aquaculture 2: 31–35.

[COU047C56] Speers-Roesch B Richards JG Brauner CJ Farrell AP Hickey AJR Wang YS Renshaw GMC (2012a) Hypoxia tolerance in elasmobranchs. I. Critical oxygen tension as a measure of blood oxygen transport during hypoxia exposure. J Exp Biol 215: 93–102. 22162857 10.1242/jeb.059642

[COU047C57] Speers-Roesch B Brauner CJ Farrell AP Hickey AJR Renshaw GMC Wang YS Richards JG (2012b) Hypoxia tolerance in elasmobranchs. II. Cardiovascular function and tissue metabolic responses during progressive and relative hypoxia exposures. J Exp Biol 215: 103–114. 22162858 10.1242/jeb.059667

[COU047C58] Steffensen JF (1989) Some errors in respirometry of aquatic breathers: how to avoid and correct them. Fish Physiol Biochem 6: 49–59. 24226899 10.1007/BF02995809

[COU047C59] Steffensen JF Johansen K Bushnell PG (1984) An automated swimming respirometer. Comp Biochem Physiol A Physiol 79: 437–440.

[COU047C60] Tresguerres M Parks SK Salazar E Levin LR Goss GG Buck J (2010) Bicarbonate-sensing soluble adenylyl cyclase is an essential sensor for acid/base homeostasis. Proc Natl Acad Sci USA 107: 442–447. 20018667 10.1073/pnas.0911790107 PMC2806762

[COU047C61] Turner JD Wood CM Höbe H (1983) Physiological consequences of severe exercise in the inactive benthic flathead sole (*Hippoglossoides elassodon*): a comparison with the active pelagic rainbow trout (*Salmo gairdneri*). J Exp Biol 104: 269–288.

[COU047C62] Wells R McIntyre R Morgan A Davie P (1986) Physiological stress responses in big gamefish after capture: observations on plasma chemistry and blood factors. Comp Biochem Physiol A Physiol 84: 565–571. 10.1016/0300-9629(86)90366-x 2874936

[COU047C63] Wise G Mulvey JM Renshaw GM (1998) Hypoxia tolerance in the epaulette shark (*Hemiscyllium ocellatum*). J Exp Zool 281: 1–5.

[COU047C64] Wood CM McMahon B McDonald D (1977) An analysis of changes in blood pH following exhausting activity in the starry flounder, *Platichthys stellatus*. J Exp Biol 69: 173–185. 908908 10.1242/jeb.69.1.173

[COU047C65] Wood CM Pärt P Wright PA (1995) Ammonia and urea metabolism in relation to gill function and acid–base balance in a marine elasmobranch, the spiny dogfish (*Squalus acanthias*). J Exp Biol 198: 1545–1558. 9319448 10.1242/jeb.198.7.1545

[COU047C66] Wood CM Kajimura M Bucking C Walsh PJ (2007) Osmoregulation, ionoregulation and acid–base regulation by the gastrointestinal tract after feeding in the elasmobranch (*Squalus acanthias*). J Exp Biol 210: 1335–1349. 17401117 10.1242/jeb.02736

